# Transitional B cells and CD21low in patients with ataxia-telangiectasia

**DOI:** 10.1186/1939-4551-8-S1-A63

**Published:** 2015-04-08

**Authors:** Danielli Christinni Bichuetti-Silva, Camila Teles Machado Pereira, Nadijane Ferreira, Reinaldo Salomão, Milena Karina Coló Brunialti, Beatriz Costa Carvalho

**Affiliations:** 1 Federal University of Sao Paulo, Brazil

## Background

We aim to evaluate the proportion of transitional immature and CD21low B cells in patients with Ataxia-telangiectasia (AT), a complex disease with humoral and cellular immune dysfunction.

## Methods

Blood samples were obtained from 18 AT patients and 15 age-sex-matched controls (C). This study was approved by the Medical Ethic Committee of the Federal University of Sao Paulo. Total numbers of T, B, and NK cells were enumerated from whole blood samples using TruCount Tubes. Peripheral blood mononuclear cells (PBMC) were cryopreserved, thawed and stained with conjugated monoclonal antibodies: anti-CD19-PerCP anti-CD3-APCCy7, anti-CD24-PE, anti-CD21-APC, anti-CD38-PECy7. Five-color flow cytometric immunophenotyping was performed on a BD LSRFortessa (BD Biosciences), and data were analyzed with FlowJo software (TreeStar, Stanford University, CA). Transitional B cells were characterized as CD3^-^CD19^+^CD24^hi^CD38^hi^, and CD21low B cells as CD3^-^CD19^+^CD21^lo^CD38^lo^. Statistical analysis was performed with SPSS 20.0 and STATA 12, and a significance threshold of <0.05 was used.

## Results

From 18 patients, 15 were male and 3 female, aged from 5 to 25 years old. There were 3 pairs of siblings. Consanguinity was present between 2 parents. Ten of them are being treated with immunoglobulin replacement therapy. One of them had recovered from a neoplastic hematologic disease. The total number of lymphocytes was reduced in AT patients (928 - 4579 cel/mm^3^) compared to controls (1646 – 6601 cel/mm^3^) (p=0.001). Total CD3^+^ (AT = 1163.8 cel/mm^3^; C= 2247.2 cel/mm^3^; p<0.001), CD4^+^ (AT = 531.4 cel/mm^3^; C= 1153.3 cel/mm^3^; p<0.001) and CD8^+^ (AT = 507.6 cel/mm^3^; C= 880.3 cel/mm^3^; p=0.007) numbers were decreased as well.B cells counts also showed a reduction compared with healthy controls (AT = 118.7 cel/mm^3^; C= 649.3 cel/mm^3^; p<0.001). By contrast, natural killer numbers were increased (AT = 583.9 cel/mm^3^; C= 357.3 cel/mm^3^; p=0.04). Proportion of Transitional B cells was reduced compared with those seen in healthy control subjects (AT= 2.2%; C= 7.3%; p=0.001). On the other hand, the CD21low B Cells showed an increased proportion (AT= 25%; C=4.9%; p<0.001).

## Conclusions

Patients with AT had disturbed B-cell homeostasis as evidenced by low transitional B cells and a large proportion CD21low B cells.

